# Dietary Intake of Protein from Different Sources and Weight Regain, Changes in Body Composition and Cardiometabolic Risk Factors after Weight Loss: The DIOGenes Study

**DOI:** 10.3390/nu9121326

**Published:** 2017-12-06

**Authors:** Marleen A. van Baak, Thomas M. Larsen, Susan A. Jebb, Alfredo Martinez, Wim H. M. Saris, Teodora Handjieva-Darlenska, Anthony Kafatos, Andreas F. H. Pfeiffer, Marie Kunešová, Arne Astrup

**Affiliations:** 1 Department of Human Biology, NUTRIM School for Nutrition and Translational Research in Metabolism, Maastricht University Medical Centre, 6200MD Maastricht, The Netherlands; w.saris@maastrichtuniversity.nl; 2 Department of Nutrition, Exercise and Sports (NEXS), Faculty of Science, University of Copenhagen, 2200 Copenhagen, Denmark; tml@life.ku.dk (T.M.L.); ast@life.ku.dk (A.A.); 3 Nuffield Department of Primary Care Health Sciences, University of Oxford, Oxford OX2 6GG, UK; susan.jebb@phc.ox.ac.uk; 4 Department of Physiology and Nutrition, University of Navarra, 31008 Pamplona, Spain; jalfmtz@unav.es; 5 Department of Pharmacology and Toxicology, Faculty of Medicine, Medical University, 1000 Sofia, Bulgaria; teodorah@abv.bg; 6 Department of Social Medicine, Preventive Medicine & Nutrition Clinic, University of Crete, 71003 Heraklion, Greece; kafatos@med.uoc.gr; 7 Department of Clinical Nutrition, German Institute of Human Nutrition Potsdam-Rehbrücke, 14558 Nuthetal, Germany; afhp@dife.de; 8 Obesity Management Center, Institute of Endocrinology, 11694 Prague, Czech Republic; mkunesova@endo.cz

**Keywords:** weight loss maintenance, diet, protein sources, cardiometabolic risk factors, obesity, plant protein, animal protein, meat protein, cereal protein

## Abstract

An increase in dietary protein intake has been shown to improve weight loss maintenance in the DIOGenes trial. Here, we analysed whether the source of the dietary proteins influenced changes in body weight, body composition, and cardiometabolic risk factors during the weight maintenance period while following an energy-restricted diet. 489 overweight or obese participants of the DIOGenes trial from eight European countries were included. They successfully lost >8% of body weight and subsequently completed a six month weight maintenance period, in which they consumed an ad libitum diet varying in protein content and glycemic index. Dietary intake was estimated from three-day food diaries. A higher plant protein intake with a proportional decrease in animal protein intake did not affect body weight maintenance or cardiometabolic risk factors. A higher plant protein intake from non-cereal products instead of cereal products was associated with benefits for body weight maintenance and blood pressure. Substituting meat protein for protein from other animal sources increased insulin and HOMA-IR (homeostasis model assessment of insulin resistance). This analysis suggests that not only the amount of dietary proteins, but also the source may be important for weight and cardiometabolic risk management. However, randomized trials are needed to test the causality of these associations.

## 1. Introduction

Diet composition is known to affect health. Evidence from randomized clinical trials suggests that an increased dietary protein content helps to increase weight loss and limit weight regain in overweight and obese individuals [1,2], although observational studies do not always support a negative association between dietary protein content and prospective weight gain [3,4]. In addition, the beneficial effects of increased protein consumption on blood pressure, HDL (high density lipoprotein) cholesterol, fasting insulin, and triglyceride concentrations have been reported in a meta-analysis of human intervention studies [1]. Much less is known about the importance of the sources from which these proteins are derived for these beneficial effects. A review on the effect of proteins from different sources on body composition concluded that the evidence for differences among protein sources is currently inconclusive [5]. In contrast, two large observational studies reported that long-term weight gain was associated with a higher intake of proteins from animal sources [3,6]. Another review focusing on protein sources in relation to coronary heart disease and underlying risk factors did not reveal clear differences among protein sources [7]. On the other hand, the Nurses’ Health Study suggested that consuming poultry, fish, low-fat dairy, or nuts instead of red meat would lower the risk of coronary heart disease and stroke [8,9]. A review on the role of dietary proteins in blood pressure (BP) control concluded that there may be a small beneficial effect of protein on BP, especially for plant protein [10]. A higher animal protein intake was associated with a higher risk of type 2 diabetes in the EPIC-NL (European Prospective Investigation Into Cancer and Nutrition–Netherlands) study, whereas no association with plant protein intake was found [11].

Given the paucity and inconsistency of data on protein intake from various sources on body weight and cardiometabolic risk, we took the opportunity to conduct a secondary analysis of data from the randomized controlled DIOGenes trial, which investigated the role of dietary protein content and glycemic index (GI) on weight loss maintenance and cardiometabolic risk factors in overweight and obese families [12,13]. We focused our analyses on protein sources from food groups that made an important contribution to the diet. In this exploratory secondary analysis, we therefore studied whether substituting animal protein for plant protein, or cereal-protein for non-cereal protein, or meat for other animal protein, would affect these parameters.

## 2. Materials and Methods

Data were collected in the context of the DIOGenes randomized clinical trial that was executed in eight European countries between 2005 and 2009. The aim of the DIOGenes trial was to investigate the effect of diet composition on weight loss maintenance in families with one or both parents overweight or obese, with a focus on dietary protein content and glycemic index. The design and main results of the trial have been published previously [12,13]. The study was conducted according to the Declaration of Helsinki, and was approved by the Medical Ethical Committee of the University of Copenhagen (KF01-267787 IHE 4-1-2.0091 dd. 23-03-2006) and the local ethical committees in the respective countries. All of the subjects gave written informed consent before starting their participation in the study.

### 2.1. Subjects

All of the adult participants of the DIOGenes trial who had completed the six-month dietary intervention phase after the successful completion of an initial eight-week weight loss phase (i.e., attained weight loss ≥8%) were included. Details on recruitment and exclusion criteria have been provided in a previous paper [13]. Subjects were healthy, had a BMI (Body Mass Index) ≥ 27 kg/m^2^, were below 65 years of age and had at least one child aged 5–18 years living in their household.

### 2.2. Study Design

After inclusion into the study, subjects started an eight-week low-calorie diet (LCD) period. If ≥8% of initial body weight was lost after eight weeks, families were randomized to one of five *ad libitum* maintenance diets for 26 weeks. 773 adult participants were randomized. The five diet groups were a high protein/high GI group, a low protein/high GI group, a high protein/low GI group, low protein/low GI group and a group that followed the recommended diet in each of the countries. The low protein groups were advised a diet with a protein content between 10 and 15% of energy intake (% energy), the high protein groups a protein content between 23 and 28 energy %. The targeted difference in dietary GI between the low and high GI groups was 15 glycemic-index units. All of the diets advised a fat intake of <30 energy %. The aim of this 26-week intervention period was to maintain body weight loss. Dietary instructions on the *ad libitum* diets were provided by trained dietitians, as described previously [14]. In addition, two of the centres (Copenhagen and Maastricht) provided the foods free of charge to the participants from laboratory supermarkets [13].

### 2.3. Procedures

Before the start of the weight loss phase (baseline), subjects filled in a three-day food diary (two week days and one weekend day). Subsequently, they came for a clinical investigation day (CID1 = baseline) to the research centers. Measurements on a CID included anthropometric and blood pressure measurements, a fasting blood sample and an oral glucose tolerance test with blood collection. These clinical investigations were repeated at the end of the eight-week weight loss (WL) period (CID2), and at the end of the 26-week weight maintenance (WM) period (CID3). Additional three-day food diaries were collected after four weeks, and in the last week (week 26) of the WM phase. The 26-week maintenance phase was completed by 556 participants, in 489 of those data on dietary intake were also available (at week 4 (*N* = 410) or week 26 (*N* = 443) or both (*N* = 364)). These 489 subjects were included in the final analysis. For the clinical measurements the same standard operating procedures were used in all centers and all of the blood samples were analyzed in one laboratory.

### 2.4. Clinical Examinations, Oral Glucose Tolerance Test (OGTT) and Blood Analysis

Body weight, waist circumference, body composition, and blood pressure were measured, as described previously [13]. Subsequently, an OGTT was performed [13]. The Matsuda index [15], a measure of insulin sensitivity in stimulated condition derived from fasting glucose and insulin and the glucose and insulin responses during the OGTT, was calculated. From the fasting glucose and the insulin concentrations, the HOMA-IR index, a measure of fasting insulin sensitivity, was calculated as: (fasting insulin (mIU/L) × fasting glucose (mmol/L))/22.5 [16]. In fasting blood samples, plasma adiponectin concentration and serum concentrations of glucose, insulin, total cholesterol, HDL cholesterol, triglycerides, and C-reactive protein (CRP) were measured, as described previously [13,17].

### 2.5. Dietary Records

All of the families were provided with household weighing scales. Dietitians instructed the subjects on how to complete the three-day food record on three consecutive days, including two weekdays and one weekend day. Participants were instructed to weigh all of their foods and left-overs and to provide brand names and details on cooking and processing where relevant. When weighing was not possible (e.g., when eating out), subjects were instructed to record food intake in household measures (cups, glasses, table spoons, etc.).

### 2.6. Dietary Analysis

Energy and nutrient intakes and glycemic index (GI) were calculated using national food and nutrient databases [18,19,20,21,22,23] where these were available (UK, Denmark, The Netherlands, Germany, and Czech Republic). The UK data were used by the Spanish, Bulgarian, and Greek centres. Information from food labels, and relevant diet composition data that was provided by fast food restaurants regarding their own products were also included. Available carbohydrate is described as monosaccharide equivalents (MSE) in the UK database [19] and was therefore slightly higher compared with other databases [20,21,22,23], which use available carbohydrate values. To adjust for this difference the carbohydrate values from the UK database were standardized using the formula below, where starch, monosaccharide, and disaccharide values were all available in the food composition database:

Available carbohydrate = (starch_(MSE)_/1.1) + (disaccharide_(MSE)_/1.05) + (monosaccharide_(MSE)_)

Fibre values from the UK database were adjusted for compatibility with the other food and nutrient databases. GI values were added to the tables using a procedure described in more detail elsewhere [18]. Intake of protein and GI of the diets were calculated. For the analysis of the different types of protein, all of the foods in the database were assigned to food groups. From each amount of a food consumed within a food group, the protein content (in grams) was assigned to the food group. Thus, protein intake from each food group could be determined. For protein intake, the following food groups were distinguished: (a) cereal protein intake (including protein from breads and rolls, breakfast cereals, cereal bars, flour, pasta and pasta dishes, bakery products, rice, and other cereal products); (b) non-cereal plant protein intake (including protein from nuts, pulses, vegetables, starchy roots and potatoes, fruits, non-dairy beverages); (c) red meat protein intake; (d) poultry protein intake; (e) fish protein intake; (f) dairy protein intake (including protein from milk, cheese, cream, yoghurt, ice cream and other milk products); and, (g) animal protein intake from other sources (including eggs, offal). Total plant protein intake was the combination of food groups (a) and (b), total animal protein intake was the combination of groups (c) to (g) and total meat protein as the sum of (c) and (d). If a food or dish contained a mixture of proteins from different sources, an estimation of the composition was made based on the ingredients. This was the case for 21% of protein intake at baseline and 16% and 18% during follow-up. Dietary intake during the 26-week intervention was calculated as the mean intake reported in the diaries at week 4 and week 26. If one of them was missing, then the values of the remaining diary were taken to reflect intake during the intervention.

### 2.7. Statistical Analysis

Data on 489 subjects were available for analysis. Because of missing values for some of the measurements, the numbers for the different parameters may be lower. Data were analyzed by regression analysis. Dependent variables were the changes in anthropometrics, blood pressure, and metabolic parameters during the weight maintenance period. The basic model included dietary protein and fat intake (as % of total energy intake), dietary GI, and fibre intake (g/1000 kcal) as dietary factors. In addition, the type of centre, gender, BMI at randomization, and the change in the dependent variable during the weight loss (WL) phase as independent variables. This model tests the effect of replacing carbohydrate by proteins in the diet on weight regain [24]. The regression coefficient (B) gives the change in weight regain (kg) with a 1 energy % change in intake from proteins at the cost of carbohydrates. The standardized regression coefficient beta denotes the number of standard deviations that the outcome will change as a result of a one standard deviation change in the predictor. Subsequently, we tested the effect of variations in the intake of animal protein (as % of total protein intake). The results of this analysis indicate whether increasing or decreasing the animal protein intake at the cost of plant protein intake (adjusting for total protein intake) had a significant effect on the dependent variable. Additionally, we tested the effect of variations in cereal protein intake (as % of total plant protein intake, adjusting for total and animal protein intake). The result of this analysis indicates whether increasing or decreasing cereal protein intake at the cost of other plant proteins affects the dependent variable. We did similar analyses for the effects of meat protein (adjusting for total protein and animal protein intake). Statistical analyses were performed with SPSS Statistics version 20 (IBM, Armonk, NY, USA). A *p* value of < 0.05 was considered to be statistically significant. In order to reduce the risk of false positive findings but on the other hand not to lose potentially relevant information, we set the significance level at a *p* value < 0.010 in the regression analyses.

## 3. Results

489 subjects were included in the analysis. Age was 42.3 ± 0.3 years (mean ± SE, standard error), BMI was 34.3 ± 0.2 kg/m^2^ and 167 subjects were male and 322 female. Further clinical characteristics are shown in Table 1. The WL phase induced significant decreases in body weight, body fat percentage, BMI, waist circumference, blood pressure, blood lipids, fasting glucose and insulin, HOMA-IR index and CRP, and significantly increased the Matsuda index. Only the adiponectin concentration did not change significantly (Table 1). During the WM phase significant increases from the end of the WL phase were found for all variables except the Matsuda index and CRP, which decreased significantly (Table 1).

Diet composition was significantly different between baseline and the WM phase (Table 2). GI was reduced and the total protein intake (as % of energy intake) was increased during the WM phase as compared with the baseline. The percentage of total protein intake from animal sources increased slightly with a proportional decrease in the intake of protein from plant sources. During WM, the proportion of cereal protein (as % of plant protein intake) and red meat protein (as % of animal protein intake) was lower than at baseline, the intakes of dairy and fish (as % of animal protein intake) were significantly higher (Table 2).

### 3.1. Total Protein Intake

A higher protein intake was associated with a significantly less regain of body weight and less of an increase in the total cholesterol and fasting glucose concentration (all *p* < 0.01) (Table 3). The increase in total cholesterol was no longer statistically significant after entering weight change over the weight maintenance period in the model, suggesting it is at least partly mediated by the weight change during the WM phase. On the other hand, the change in fasting glucose remained statistically significant after adjusting for weight change (Table 3).

### 3.2. Substituting Plant Proteins for Animal Proteins

Including animal protein intake (as % of total protein intake) into the model did not significantly add to the explained variance for any of the variables studied (Table 4). This suggests that substituting plant proteins for animal proteins is not associated with beneficial effects on any of the studied variables.

### 3.3. Substituting Cereal Protein for Non-Cereal Plant Proteins

When cereal protein intake (as % of plant protein intake) was added to the model, a higher intake of cereal protein at the cost of non-cereal plant protein, was associated with a larger increase in body weight and systolic BP (all *p* < 0.010) (Table 5). When the change in body weight during the WM phase was included in the model, the effect on systolic BP remained statistically significant (*p* = 0.003), which suggests that it was independent of concurrent changes in body weight.

### 3.4. Substituting Meat Protein for Proteins from Other Animal Sources

A substitution of total meat protein intake for non-meat animal protein showed a significant positive association with the changes in fasting insulin and HOMA-IR (both *p* < 0.010) (Table 6). Adding the weight change during the WM phase to the models did not affect this association, suggesting that the associations were not dependent on concurrent weight changes. Substituting dairy protein for non-dairy animal protein or fish protein for non-fish animal protein was not associated with statistically significant changes in any of the studied variables (Supplemental Tables S1–S4).

## 4. Discussion

Many studies have shown that dietary protein content may play a role in weight management, with the DIOGenes trial being one of them [12,25]. In this study, we examined the role of different sources of dietary proteins (independent of total protein intake) on the change in body weight and body composition, and on the cardiometabolic risk factors during the weight maintenance phase of the Diogenes trial. Our initial analysis confirmed the main outcome of the DIOGenes RCT (randomized controlled trial), i.e., less weight regain on a diet with higher protein content [12,25], although we did not analyze the data according to the diet groups into which subjects were randomized, but based on self-reported dietary intake of protein. The regression model indicates that a 5% higher protein intake would be associated with approximately 0.85 kg less weight regain over six months in this study.

### 4.1. Animal vs. Plant Protein

In our study substitution of animal protein for plant protein (adjusting for total protein intake) had no significant effects on the changes in any of the studied variables. Randomized trials comparing the effects of plant and animal protein-rich diets on cardiometabolic risk factors are scarce. One trial compared modified DASH (Dietary Approaches to Stop Hypertension) diets enriched in either plant or animal protein during weight loss and weight maintenance and concluded that diet composition did not affect weight changes or metabolic syndrome criteria differently [26]. Some trials are available comparing selected plant and animal proteins. A recent study [27] explored the effect of isocaloric whey, soy, and maltodextrin supplements on weight regain after weight loss and found no significant differences. The effects of soy and whey protein provided as supplements on body weight and body composition have also been compared in free-living overweight or obese individuals without prior weight loss. The whey protein supplemented group reached a significantly lower body weight than the group that took isocaloric maltodextrin supplements, but there was no significant difference with the soy protein supplemented group after five weeks [28]. Several studies have demonstrated an inverse relationship between the intake of protein from plant sources and BP [10], although evidence from RCT’s is lacking [10,29]. Also, in these studies, it is unclear whether the effect of plant protein is independent of the effect of total protein intake. In our study we found no evidence for an association between the ratio of plant to animal protein intake and BP changes, independent of protein intake. Soy protein has been shown to have more beneficial effects on blood lipids than milk protein in healthy adults without hypercholesterolemia [30]. No clear associations have been found for animal or plant protein intake and the risk of coronary heart disease and stroke [9,31,32]. High intakes of animal protein, but not plant protein, have been associated with an increased diabetes risk [11,33,34]. A similar association was found for total protein intake [11,34]. In our study, we found no indications that substituting plant protein for animal protein would have an effect on the change in glucose homeostasis. An analysis of data from the Nurses Health Study showed that substituting plant protein for animal protein reduced cardiovascular mortality, especially when red meat was replaced, in individuals with at least one unhealthy lifestyle factor, including obesity [35].

### 4.2. Substituting Cereal for Non-Cereal Plant Proteins

A higher cereal protein intake, without changes in total plant and animal protein intake, was associated with unfavorable effects on body weight gain and changes in systolic blood pressure in our study population. This suggests that replacing cereal protein by an equal amount of non-cereal plant protein, e.g., from pulses, vegetables, nuts, fruits, and potatoes, may have beneficial effects on weight regain and associated changes in those cardiometabolic risk factors. A meta-analysis of randomized controlled trials comparing diets in which whole pulses were exchanged for other dietary components showed that dietary pulses lead to modest weight loss. Pulses may therefore explain (part of) this effect [36]. In addition, evidence suggests that the consumption of refined grains is associated with weight gain [37]. It is unclear whether the effects are related to differences in the protein or amino acid composition of the protein fractions of cereal and non-cereal foods or to differences in other aspects of these foods, such as the presence of bioactive compounds, which may affect satiety or energy expenditure. So far, no studies have directly compared the exchange between cereal and non-cereal protein, or cereal and non-cereal foods. Differences in GI and fiber are unlikely to play a role, because these factors were included in the model.

### 4.3. Substituting Meat for Other Animal Proteins

As discussed by Comerford and Pasin, animal proteins from different sources have different qualities [38], which complicate the research, because all of the possible combinations of types of animal proteins, even within groups such as dairy, fish, and meat, should ideally be taken into account. In this study, we found that higher consumption of meat instead of other animal proteins, such as dairy, fish, and eggs, did not affect weight regain but was associated with a larger increase in fasting insulin and HOMA-IR during the weight maintenance phase. Which dietary factor(s) is responsible for these associations is not clear. When we investigated substituting red meat, poultry, fish, and dairy for other animal proteins separately, we did not find significant associations with the increase in fasting insulin and HOMA-IR (Supplementary Tables S1–S4). Red meat consumption has been associated with the risk of type 2 diabetes, but this association appears to be mainly due to processed red meat consumption, and is therefore most likely related to non-protein factors [33]. In randomized diet interventions in overweight adults comparing beef or chicken as the primary protein source, no differences in body weight or fat loss were found [39,40,41].

The study reported here has certain limitations that need to be acknowledged. Although the DIOGenes trial was a randomized controlled trial, for the analysis reported here the results were analyzed as an observational study. Therefore, the strengths of the original RCT study design have been lost. On the other hand, this analysis allowed us to take variations in more dietary factors than the main factors in the DIOGenes trial (protein intake and GI) into account in a considerable number of subjects with a large number of clinical measurements. A weak point in all of the dietary analysis studies is that we have to rely on self-reported data of which the quality is known to be a problem. Although our analyses focused on the protein intake from various food groups, the interpretation of the results is complicated since we cannot exclude that it is not the protein intake from certain sources, but rather the complete foods from these sources that are responsible for the effects that were found. Misclassification of protein sources also cannot be ruled out, especially for foods with mixed proteins, which comprised 16–21% of protein intake. Although we adjusted for a number of the most important interfering factors (such as fibre, fat, and GI), residual confounding cannot be excluded. In addition, we cannot rule out that the associations are confounded by other lifestyle factors, such as physical activity, impaired or less sleep, stress, socioeconomic status, and education.

## 5. Conclusions

In summary, we found no indications that a higher plant protein intake with a proportional decrease in animal protein intake affects body weight maintenance after weight loss or changes in cardiometabolic risk factors during this period. A higher plant protein intake from non-cereal products was associated with benefits for body weight maintenance and systolic blood pressure. Meat protein consumption instead of protein from other animal sources was associated with increases in fasting insulin and HOMA-IR. This analysis therefore suggests that not only the amount of dietary proteins, but also the source, may be important in weight and cardiometabolic risk management. However, randomized trials are needed to test whether these associations are indeed causal.

## Figures and Tables

**Table 1:** Characteristics of the participants at the three clinical investigation days (CID1 = baseline; CID2 = end of weight loss phase; CID = end of weight maintenance phase) ^1^.

Variable ^2^	CID1	CID2	CID3	*p* Value ^3^	*p* Value ^4^
Body weight (kg)	99.5 ± 0.8	88.3 ± 0.7	88.8 ± 0.7	<0.001	0.031
Body fat (%)	40.3 ± 0.4	36.2 ± 0.5	35.2 ± 0.4	<0.001	<0.001
BMI (kg/m^2^)	34.3 ± 0.2	30.5 ± 0.2	30.6 ± 0.2	<0.001	0.039
Waist circumference (cm)	107.4 ± 0.6	97.1 ± 0.6	97.7 ± 0.6	<0.001	0.048
SBP (mm Hg)	125.4 ± 0.7	117.5 ± 0.6	122.2 ± 0.6	<0.001	<0.001
DBP (mm Hg)	77.6 ± 0.5	72.3 ± 0.4	74.5 ± 0.5	<0.001	<0.001
Totalcholesterol (mmol/L)	4.85 ± 0.05	4.15 ± 0.04	4.90 ± 0.04	<0.001	<0.001
HDL cholesterol (mmol/L)	1.23 ± 0.02	1.15 ± 0.01	1.38 ± 0.02	<0.001	<0.001
LDL cholesterol (mmol/L)	3.01 ± 0.04	2.52 ± 0.04	2.98 ± 0.04	<0.001	<0.001
Glucose (mmol/L)	5.09 ± 0.03	4.80 ± 0.02	4.93 ± 0.02	<0.001	<0.001
Insulin (mIU/L)	12.42 ± 0.59	8.33 ± 0.49	9.49 ± 0.50	<0.001	0.001
HOMA IR index	3.36 ± 0.17	2.13 ± 0.14	2.50 ± 0.14	<0.001	0.001
Matsuda index	4.87 ± 0.15	7.23 ± 0.19	6.80 ± 0.19	<0.001	0.011
CRP (mg/L)	3.65 ± 0.15	2.65 ± 0.12	2.37 ± 0.11	<0.001	0.009
Adiponectin (mg/L)	9.18 ± 0.20	9.38 ± 0.18	10.87 ± 0.21	0.230	<0.001

^1^ Values are mean ± SEM, (Standard Error of Mean); ^2^ Repeated measures ANOVA (CID1 vs. CID2 vs. CID3) *p* < 0.001 for all variables; ^3^ *p* value of post-hoc comparison CID1 vs. CID2; ^4^ *p* value of post-hoc comparison CID2 vs. CID3. SBP: systolic blood pressure. DBP: diastolic blood pressure.

**Table 2:** Dietary intakes at baseline during the weight maintenance phase ^1^.

Variable	Baseline	Weight Maintenance	*p* Value ^2^
Energy intake (kJ/day)	9380 ± 143	6668 ± 101	<0.001
Total protein intake (% energy)	16.9 ± 0.2	19.9 ± 0.2	<0.001
GI (GI units)	61.1 ± 0.2	58.5 ± 0.2	<0.001
CHO intake (% energy)	44.6 ± 0.4	48.1 ± 0.4	<0.001
Fat intake (% energy)	36.2 ± 0.3	30.5 ± 0.3	<0.001
Fiber intake (g/1000 kJ)	2.14 ± 0.04	3.12 ± 0.05	<0.001
Animal protein intake (% of total protein intake)	61.2 ± 0.6	62.3 ± 0.6	0.036
Plant protein intake (% of total protein intake)	38.8 ± 0.6	37.7 ± 0.6	0.035
Cereal protein intake (% of plant protein intake)	68.9 ± 0.9	61.3 ± 0.8	<0.001
Red meat protein intake (% of animal protein intake)	37.0 ± 1.0	28.0 ± 0.9	<0.001
Dairy protein intake (% of animal protein intake)	31.1 ± 0.8	34.0 ± 0.8	0.003
Poultry meat protein intake (% of animal protein intake)	12.7 ± 0.8	14.8 ± 0.6	0.092
Fish protein intake (% of animal protein intake)	8.1 ± 0.6	14.0 ± 0.7	<0.001

^1^ Values are mean ± SEM; ^2^ *p* value of difference between baseline and weight maintenance. CHO: Carbohydrate.

**Table 3:** Regression coefficients (B), their standard error (SE) and the standardized regression coefficients (Beta) for the associations between changes in anthropometric variables, blood pressure and metabolic factors during the weight maintenance phase and total protein intake (% of energy intake) ^1^.

Variable	B	SE	Beta	*p* Value
Body weight (kg)	−0.169	0.053	−0.142	**0.001**
Body fat (%)	−0.076	0.049	−0.084	0.120
Waist circumference (cm)	−0.027	0.065	−0.019	0.676
Systolic BP (mm Hg)	−0.178	0.114	−0.066	0.120
Diastolic BP (mm Hg)	−0.136	0.076	−0.078	0.074
Total cholesterol (mmol/L)	−0.018	0.007	−0.109	**0.005**
HDL cholesterol (mmol/L)	−0.004	0.002	−0.084	0.050
LDL cholesterol (mmol/L)	−0.011	0.006	−0.078	0.045
Triglycerides (mmol/L)	−0.009	0.004	−0.091	0.031
Fasting glucose (mmol/L)	−0.014	0.004	−0.136	0.001 *
Fasting insulin (mIU/L)	−0.132	0.072	−0.098	0.069
HOMA-IR	−0.037	0.023	−0.080	0.107
Matsuda index	0.035	0.030	0.052	0.245
CRP (mg/L)	−0.005	0.021	−0.010	0.826
Adiponectin (mg/L)	−0.033	0.034	−0.040	0.327

^1^ Regression models also included BMI at randomization, changes in the dependent variables during the weight loss phase, gender, type of centre, GI, dietary fat intake (as % of energy intake) and fibre intake (g/1000 kcal); * remains significant after adjusting for weight change during WM.

**Table 4:** Regression coefficients (B), their standard error (SE) and the standardized regression coefficients (Beta) for the associations between changes in anthropometric variables, blood pressure and metabolic factors during the weight maintenance phase and animal protein intake (% of total protein intake) ^1^.

Variable	B	SE	Beta	*p* Value
Body weight (kg)	0.040	0.032	0.086	0.213
Body fat (%)	0.075	0.029	0.216	0.011
Waist circumference (cm)	0.040	0.041	0.073	0.321
Systolic BP (mm Hg)	−0.012	0.070	−0.011	0.866
Diastolic BP (mm Hg)	0.081	0.046	0.121	0.081
Total cholesterol (mmol/L)	0.008	0.004	0.126	0.043
HDL cholesterol (mmol/L)	0.000	0.001	0.021	0.766
LDL cholesterol (mmol/L)	0.008	0.003	0.142	0.021
Triglycerides (mmol/L)	−0.001	0.003	−0.021	0.759
Fasting glucose (mmol/L)	0.000	0.003	0.006	0.933
Fasting insulin (mIU/L)	0.004	0.046	0.007	0.939
HOMA-IR	0.002	0.015	0.010	0.898
Matsuda index	−0.005	0.019	−0.019	0.790
CRP (mg/L)	−0.013	0.013	−0.069	0.335
Adiponectin (mg/L)	−0.035	0.021	−0.112	0.088

^1^ Regression models also included BMI at randomization, changes in the dependent variables during the weight loss phase, gender, type of centre, dietary protein intake (as % of energy intake), GI, dietary fat intake (as % of energy intake) and fibre intake (g/1000 kcal).

**Table 5:** Regression coefficients (B), their standard error (SE) and the standardized regression coefficients (Beta) for the associations between cereal protein intake (% of total plant protein intake) and changes in anthropometric variables, blood pressure and metabolic factors during the weight maintenance phase ^1^.

Variable	B	SE	Beta	*p* Value
Body weight (kg)	0.045	0.014	0.150	0.001
Body fat (%)	0.031	0.013	0.146	0.014
Waist circumference (cm)	0.045	0.017	0.127	0.011
Systolic BP (mm Hg)	0.113	0.030	0.166	0.000 *
Diastolic BP (mm Hg)	0.040	0.020	0.092	0.049
Total cholesterol (mmol/L)	−0.001	0.002	−0.030	0.475
HDL cholesterol (mmol/L)	0.000	0.001	−0.016	0.731
LDL cholesterol (mmol/L)	−0.002	0.002	−0.062	0.143
Triglycerides (mmol/L)	0.003	0.001	0.108	0.018
Fasting glucose (mmol/L)	0.001	0.001	0.040	0.389
Fasting insulin (mIU/L)	−0.022	0.020	−0.065	0.267
HOMA-IR	−0.005	0.006	−0.048	0.380
Matsuda index	0.003	0.008	0.016	0.745
CRP (mg/L)	−0.012	0.006	−0.106	0.032
Adiponectin (mg/L)	−0.018	0.009	−0.090	0.046

^1^ Regression models also included BMI at randomization, changes in the dependent variables during the weight loss phase, gender, type of centre, dietary protein intake (as % of energy intake), animal protein intake (as % of total protein intake), GI, dietary fat intake (as % of energy intake) and fibre intake (g/1000 kcal); * remains significant after adjusting for weight change during WM. LDL: low density lipoprotein.

**Table 6:** Regression coefficients (B), their standard error (SE) and the standardized regression coefficients (Beta) for the associations between changes in anthropometric variables, blood pressure, and metabolic factors during the weight maintenance phase and total meat protein intake ^1^.

Variable	B	SE	Beta	*p* Value
Body weight (kg)	0.023	0.014	0.073	0.105
Body fat (%)	0.015	0.013	0.065	0.260
Waist circumference (cm)	0.015	0.017	0.042	0.382
Systolic BP (mm Hg)	−0.035	0.030	−0.050	0.247
Diastolic BP (mm Hg)	−0.035	0.020	−0.079	0.081
Total cholesterol (mmol/L)	0.001	0.002	0.021	0.596
HDL cholesterol (mmol/L)	0.000	0.001	−0.005	0.908
LDL cholesterol (mmol/L)	0.001	0.001	0.026	0.512
Triglycerides (mmol/L)	0.000	0.001	−0.001	0.980
Fasting glucose (mmol/L)	0.001	0.001	0.029	0.507
Fasting insulin (mIU/L)	0.064	0.019	0.178	0.001 *
HOMA-IR	0.017	0.006	0.138	0.006 *
Matsuda index	−0.010	0.008	−0.057	0.207
CRP (mg/L)	−0.007	0.006	−0.059	0.201
Adiponectin (mg/L)	0.012	0.009	0.056	0.185

^1^ Regression models also included BMI at randomization, changes in the dependent variables during the weight loss phase, gender, type of centre, dietary protein intake (as % of energy intake), animal protein intake (as % of total protein intake), GI, dietary fat intake (as % of energy intake) and fibre intake (g/1000 kcal); * remains significant after adjusting for weight change during WM.

## Supplementary Materials

The following are available online at www.mdpi.com/2072-6643/9/12/1326/s1, Table S1: Regression coefficients for the associations between changes in anthropometric variables, blood pressure and metabolic factors during the weight maintenance phase and red meat protein intake; Table S2: Regression coefficients for the associations between changes in anthropometric variables, blood pressure and metabolic factors during the weight maintenance phase and dairy protein intake; Table S3: Regression coefficients for the associations between changes in anthropometric variables, blood pressure and metabolic factors during the weight maintenance phase and poultry protein intake; Table S4: Regression coefficients for the associations between changes in anthropometric variables, blood pressure and metabolic factors during the weight maintenance phase and fish protein intake.

## References

[1] Santesso N., Akl E.A., Bianchi M., Mente A., Mustafa R., Heels-Ansdell D., Schunemann H.J. (2012). Effects of higher- versus lower-protein diets on health outcomes: A systematic review and meta-analysis. Eur. J. Clin. Nutr..

[2] Wycherley T.P., Moran L.J., Clifton P.M., Noakes M., Brinkworth G.D. (2012). Effects of energy-restricted high-protein, low-fat compared with standard-protein, low-fat diets: A meta-analysis of randomized controlled trials. Am. J. Clin. Nutr..

[3] Halkjaer J., Olsen A., Overvad K., Jakobsen M.U., Boeing H., Buijsse B., Palli D., Tognon G., Du H., van der A.D. (2011). Intake of total, animal and plant protein and subsequent changes in weight or waist circumference in European men and women: The diogenes project. Int. J. Obes. (Lond.).

[4] Vergnaud A.C., Norat T., Mouw T., Romaguera D., May A.M., Bueno-de-Mesquita H.B., van der A.D., Agudo A., Wareham N., Khaw K.T. (2013). Macronutrient composition of the diet and prospective weight change in participants of the epic-panacea study. PLoS ONE.

[5] Gilbert J.A., Bendsen N.T., Tremblay A., Astrup A. (2011). Effect of proteins from different sources on body composition. Nutr. Metab. Cardiovasc. Dis..

[6] Bujnowski D., Xun P., Daviglus M.L., Van Horn L., He K., Stamler J. (2011). Longitudinal association between animal and vegetable protein intake and obesity among men in the United States: The Chicago western electric study. J. Am. Diet. Assoc..

[7] Clifton P.M. (2011). Protein and coronary heart disease: The role of different protein sources. Curr. Atheroscler. Rep..

[8] Bernstein A.M., Pan A., Rexrode K.M., Stampfer M., Hu F.B., Mozaffarian D., Willett W.C. (2012). Dietary protein sources and the risk of stroke in men and women. Stroke.

[9] Bernstein A.M., Sun Q., Hu F.B., Stampfer M.J., Manson J.E., Willett W.C. (2010). Major dietary protein sources and risk of coronary heart disease in women. Circulation.

[10] Altorf-van der Kuil W., Engberink M.F., Brink E.J., van Baak M.A., Bakker S.J., Navis G., van’t Veer P., Geleijnse J.M. (2010). Dietary protein and blood pressure: A systematic review. PLoS ONE.

[11] Sluijs I., Beulens J.W., van der A.D., Spijkerman A.M., Grobbee D.E., van der Schouw Y.T. (2010). Dietary intake of total, animal, and vegetable protein and risk of type 2 diabetes in the European prospective investigation into cancer and nutrition (epic)-nl study. Diabetes Care.

[12] Larsen T.M., Dalskov S.M., van Baak M., Jebb S.A., Papadaki A., Pfeiffer A.F., Martinez J.A., Handjieva-Darlenska T., Kunesova M., Pihlsgard M. (2010). Diets with high or low protein content and glycemic index for weight-loss maintenance. N. Engl. J. Med..

[13] Larsen T.M., Dalskov S., van Baak M., Jebb S., Kafatos A., Pfeiffer A., Martinez J.A., Handjieva-Darlenska T., Kunesova M., Holst C. (2010). The diet, obesity and genes (diogenes) dietary study in eight European countries—A comprehensive design for long-term intervention. Obes. Rev..

[14] Moore C.S., Lindroos A.K., Kreutzer M., Larsen T.M., Astrup A., van Baak M.A., Handjieva-Darlenska T., Hlavaty P., Kafatos A., Kohl A. (2010). Dietary strategy to manipulate ad libitum macronutrient intake, and glycaemic index, across eight European countries in the diogenes study. Obes. Rev..

[15] Matsuda M., DeFronzo R.A. (1999). Insulin sensitivity indices obtained from oral glucose tolerance testing: Comparison with the euglycemic insulin clamp. Diabetes Care.

[16] Wallace T.M., Levy J.C., Matthews D.R. (2004). Use and abuse of Homa modeling. Diabetes Care.

[17] Gogebakan O., Kohl A., Osterhoff M.A., van Baak M.A., Jebb S.A., Papadaki A., Martinez J.A., Handjieva-Darlenska T., Hlavaty P., Weickert M.O. (2011). Effects of weight loss and long-term weight maintenance with diets varying in protein and glycemic index on cardiovascular risk factors. Circulation.

[18] Aston L.M., Jackson D., Monsheimer S., Whybrow S., Handjieva-Darlenska T., Kreutzer M., Kohl A., Papadaki A., Martinez J.A., Kunova V. (2010). Developing a methodology for assigning glycaemic index values to foods consumed across Europe. Obes. Rev..

[19] (2002). McCance and Widdowson’s Composition of Foods integrated dataset (CoF IDS). http://www.food.gov.uk/science/dietarysurveys/dietsurveys.

[20] DTU Food, National Food Institute (2002). The Danish Food Composition Databank.

[21] Ministry of Health, Welfare and Sports (2009). NEVO-Tabel.

[22] Nutri-Science (2001). PRODI^®^.

[23] Czech Centre for Food Composition Database (2001). Czech Food Composition Database.

[24] Hu F.B., Stampfer M.J., Manson J.E., Rimm E., Colditz G.A., Rosner B.A., Hennekens C.H., Willett W.C. (1997). Dietary fat intake and the risk of coronary heart disease in women. N. Engl. J. Med..

[25] Aller E.E., Larsen T.M., Claus H., Lindroos A.K., Kafatos A., Pfeiffer A., Martinez J.A., Handjieva-Darlenska T., Kunesova M., Stender S. (2014). Weight loss maintenance in overweight subjects on ad libitum diets with high or low protein content and glycemic index: The diogenes trial 12-month results. Int. J. Obes. (Lond.).

[26] Hill A.M., Harris Jackson K.A., Roussell M.A., West S.G., Kris-Etherton P.M. (2015). Type and amount of dietary protein in the treatment of metabolic syndrome: A randomized controlled trial. Am. J. Clin. Nutr..

[27] Kjolbaek L., Sorensen L.B., Sondertoft N.B., Rasmussen C.K., Lorenzen J.K., Serena A., Astrup A., Larsen L.H. (2017). Protein supplements after weight loss do not improve weight maintenance compared with recommended dietary protein intake despite beneficial effects on appetite sensation and energy expenditure: A randomized, controlled, double-blinded trial. Am. J. Clin. Nutr..

[28] Baer D.J., Stote K.S., Paul D.R., Harris G.K., Rumpler W.V., Clevidence B.A. (2011). Whey protein but not soy protein supplementation alters body weight and composition in free-living overweight and obese adults. J. Nutr..

[29] Teunissen-Beekman K.F., van Baak M.A. (2013). The role of dietary protein in blood pressure regulation. Curr. Opin. Lipidol..

[30] Wofford M.R., Rebholz C.M., Reynolds K., Chen J., Chen C.S., Myers L., Xu J., Jones D.W., Whelton P.K., He J. (2012). Effect of soy and milk protein supplementation on serum lipid levels: A randomized controlled trial. Eur. J. Clin. Nutr..

[31] Preis S.R., Stampfer M.J., Spiegelman D., Willett W.C., Rimm E.B. (2010). Lack of association between dietary protein intake and risk of stroke among middle-aged men. Am. J. Clin. Nutr..

[32] Preis S.R., Stampfer M.J., Spiegelman D., Willett W.C., Rimm E.B. (2010). Dietary protein and risk of ischemic heart disease in middle-aged men. Am. J. Clin. Nutr..

[33] Song Y., Manson J.E., Buring J.E., Liu S. (2004). A prospective study of red meat consumption and type 2 diabetes in middle-aged and elderly women: The women’s health study. Diabetes Care.

[34] Shang X., Scott D., Hodge A.M., English D.R., Giles G.G., Ebeling P.R., Sanders K.M. (2016). Dietary protein intake and risk of type 2 diabetes: Results from the melbourne collaborative cohort study and a meta-analysis of prospective studies. Am. J. Clin. Nutr..

[35] Song M., Fung T.T., Hu F.B., Willett W.C., Longo V.D., Chan A.T., Giovannucci E.L. (2016). Association of animal and plant protein intake with all-cause and cause-specific mortality. JAMA Intern Med..

[36] Kim S.J., de Souza R.J., Choo V.L., Ha V., Cozma A.I., Chiavaroli L., Mirrahimi A., Blanco Mejia S., Di Buono M., Bernstein A.M. (2016). Effects of dietary pulse consumption on body weight: A systematic review and meta-analysis of randomized controlled trials. Am. J. Clin. Nutr..

[37] Mozaffarian D. (2016). Dietary and policy priorities for cardiovascular disease, diabetes, and obesity: A comprehensive review. Circulation.

[38] Comerford K.B., Pasin G. (2016). Emerging evidence for the importance of dietary protein source on glucoregulatory markers and type 2 diabetes: Different effects of dairy, meat, fish, egg, and plant protein foods. Nutrients.

[39] Melanson K., Gootman J., Myrdal A., Kline G., Rippe J.M. (2003). Weight loss and total lipid profile changes in overweight women consuming beef or chicken as the primary protein source. Nutrition.

[40] Mahon A.K., Flynn M.G., Stewart L.K., McFarlin B.K., Iglay H.B., Mattes R.D., Lyle R.M., Considine R.V., Campbell W.W. (2007). Protein intake during energy restriction: Effects on body composition and markers of metabolic and cardiovascular health in postmenopausal women. J. Am. Coll. Nutr..

[41] Murphy K.J., Parker B., Dyer K.A., Davis C.R., Coates A.M., Buckley J.D., Howe P.R. (2014). A comparison of regular consumption of fresh lean pork, beef and chicken on body composition: A randomized cross-over trial. Nutrients.

