# Supplementary material for: Dietary Intake of Protein from Different Sources and Weight Regain, Changes in Body Composition and Cardiometabolic Risk Factors after Weight Loss: The DIOGenes Study
Source: Nutrients. 2017 Dec 6;9(12):1326. doi: 10.3390/nu9121326 (PMC5748776; doi:10.3390/nu9121326)
Supplement: Supplementary file 1 [file nutrients-09-01326-s001.pdf]

Supplementary table S1. Regression coefficients (B) for the associations between changes in anthropometric variables, blood pressure and metabolic factors during the weight maintenance phase and red meat protein intake (as % of animal protein intake).[1]

**Variable**	**B**	**SE**	**Beta**	***p* value**
Body weight (kg)	0.040	0.014	0.129	**0.004**
Body fat (%)	0.031	0.013	0.135	0.015
Waist circumference (cm)	0.048	0.017	0.131	**0.006**
Systolic BP (mm Hg)	0.004	0.031	0.005	0.908
Diastolic BP	0.000	0.020	-0.001	0.988
Total cholesterol (mmol/L)	0.000	0.002	-0.006	0.890
HDL cholesterol (mmol/L)	0.000	0.001	-0.025	0.574
LDL cholesterol (mmol/L)	0.000	0.002	-0.005	0.896
Triglycerides (mmol/L)	0.001	0.001	0.025	0.567
Fasting glucose (mmol/L)	0.001	0.001	0.033	0.461
Fasting insulin (mIU/L)	0.032	0.020	0.087	0.112
HOMA-IR	0.006	0.006	0.049	0.336
Matsuda index	-0.003	0.008	-0.018	0.697
CRP (mg/L)	0.000	0.006	0.003	0.952
Adiponectin (mg/L)	0.003	0.009	0.016	0.701

[1] regression models also included BMI at randomization, changes in the dependent variables during the weight loss phase, gender, type of centre, dietary protein intake (as % of energy intake), animal protein intake (as % of total protein intake), GI, dietary fat intake (as % of energy intake) and fibre intake (g/1000 kcal).

Supplementary table S2. Regression coefficients (B) for the associations between changes in anthropometric variables, blood pressure and metabolic factors during the weight maintenance phase and dairy protein intake (as % of animal protein intake).[1]

**Variable**	**B**	**SE**	**Beta**	***p* value**
Body weight (kg)	-0.022	0.015	-0.067	0.143
Body fat (%)	0.009	0.014	0.037	0.511
Waist circumference (cm)	-0.015	0.019	-0.039	0.422
Systolic BP (mm Hg)	0.004	0.033	0.005	0.910
Diastolic BP (mm Hg)	0.026	0.021	0.054	0.228
Total cholesterol (mmol/L)	0.001	0.002	0.018	0.651
HDL cholesterol (mmol/L)	0.000	0.001	-0.020	0.653
LDL cholesterol (mmol/L)	0.000	0.002	0.003	0.938
Triglycerides (mmol/L)	0.002	0.001	0.074	0.090
Fasting glucose (mmol/L)	0.001	0.001	0.036	0.410
Fasting insulin (mIU/L)	-0.029	0.022	-0.074	0.186
HOMA-IR	-0.007	0.007	-0.052	0.319
Matsuda index)	0.013	0.009	0.065	0.161
CRP (mg/L)	0.007	0.006	0.050	0.280
Adiponectin (mg/L)	-0.011	0.010	-0.050	0.242

[1] regression models also included BMI at randomization, changes in the dependent variables during the weight loss phase, gender, type of centre, dietary protein intake (as % of energy intake), animal protein intake (as % of total protein intake), GI, dietary fat intake (as % of energy intake) and fibre intake (g/1000 kcal).

Supplementary table S3. Regression coefficients (B) for the associations between changes in anthropometric variables, blood pressure and metabolic factors during the weight maintenance phase and poultry protein intake (as % of animal protein intake).[1]

**Variable**	**B**	**SE**	**Beta**	***p* value**
Body weight (kg)	-0.028	0.018	-0.070	0.116
Body fat (%)	-0.031	0.017	-0.101	0.068
Waist circumference (cm)	-0.053	0.022	-0.114	0.016
Systolic BP (mm Hg)	-0.062	0.039	-0.069	0.106
Diastolic BP (mm Hg)	-0.056	0.025	-0.098	0.028
Total cholesterol (mmol/L)	0.002	0.002	0.034	0.391
HDL cholesterol (mmol/L)	0.000	0.001	0.025	0.571
LDL cholesterol (mmol/L)	0.002	0.002	0.040	0.313
Triglycerides (mmol/L)	-0.001	0.001	-0.033	0.447
Fasting glucose (mmol/L)	0.000	0.002	-0.004	0.935
Fasting insulin (mIU/L)	0.058	0.026	0.125	0.024
HOMA-IR	0.019	0.008	0.123	0.018
Matsuda index	-0.012	0.011	-0.054	0.244
CRP (mg/L)	-0.013	0.007	-0.081	0.076
Adiponectin (mg/L)	0.013	0.011	0.051	0.227

[1] regression models also included BMI at randomization, changes in the dependent variables during the weight loss phase, gender, type of centre, dietary protein intake (as % of energy intake), animal protein intake (as % of total protein intake), GI, dietary fat intake (as % of energy intake) and fibre intake (g/1000 kcal).

Supplementary table S4. Regression coefficients (B) for the associations between changes in anthropometric variables, blood pressure and metabolic factors during the weight maintenance phase and fish protein intake (as % of animal protein intake).[1]

**Variable**	**B**	**SE**	**Beta**	***p* value**
Body weight (kg)	-0.023	0.018	-0.058	0.198
Body fat (%)	-0.043	0.017	-0.141	0.010
Waist circumference (cm)	-0.034	0.022	-0.072	0.128
Systolic BP (mm Hg)	-0.010	0.039	-0.011	0.797
Diastolic BP (mm Hg)	-0.012	0.026	-0.021	0.637
Total cholesterol (mmol/L)	-0.001	0.002	-0.019	0.629
HDL cholesterol (mmol/L)	-0.001	0.001	-0.043	0.328
LDL cholesterol (mmol/L)	0.000	0.002	-0.002	0.968
Triglycerides (mmol/L)	-0.001	0.001	-0.018	0.667
Fasting glucose (mmol/L)	-0.003	0.002	-0.076	0.081
Fasting insulin (mIU/L	-0.042	0.025	-0.091	0.097
HOMA-IR	-0.008	0.008	-0.054	0.290
Matsuda index	0.013	0.010	0.056	0.217
CRP (mg/L)	0.005	0.007	0.031	0.501
Adiponectin (mg/L)	-0.011	0.011	-0.041	0.332

[1] regression models also included BMI at randomization, changes in the dependent variables during the weight loss phase, gender, type of centre, dietary protein intake (as % of energy intake), animal protein intake (as % of total protein intake), GI, dietary fat intake (as % of energy intake) and fibre intake (g/1000 kcal).
